# Evaluation of Risk Factors for Laryngeal Squamous Cell Carcinoma: A Single-Center Retrospective Study

**DOI:** 10.3389/fonc.2021.606010

**Published:** 2021-02-25

**Authors:** Qihe Zhang, Huanhuan Wang, Qin Zhao, Yuyu Zhang, Zhuangzhuang Zheng, Shiyu Liu, Zijing Liu, Lingbin Meng, Ying Xin, Xin Jiang

**Affiliations:** ^1^ Department of Radiation Oncology, The First Hospital of Jilin University, Changchun, China; ^2^ Jilin Provincial Key Laboratory of Radiation Oncology & Therapy, The First Hospital of Jilin University, Changchun, China; ^3^ NHC Key Laboratory of Radiobiology, School of Public Health, Jilin University, Changchun, China; ^4^ Key Laboratory of Pathobiology, Ministry of Education, Jilin University, Changchun, China; ^5^ Department of Hematology and Medical Oncology, Moffitt Cancer Center, Tampa, FL, United States

**Keywords:** laryngeal squamous cell carcinoma, prognostic factors, overall survival, progression-free survival, Chinese population

## Abstract

**Background:**

The survival rate of patients with laryngeal squamous cell carcinoma (LSCC) is correlated with several factors. However, the independent prognostic factors of patients with LSCC remain unclear. Thus, we sought to identify prognostic factors affecting LSCC outcomes in the Chinese population.

**Methods:**

The survival and potential prognostic factors of 211 patients with LSCC between April 2011 and July 2019 were retrospectively analyzed. Overall survival (OS) and progression free survival (PFS) were estimated by the Kaplan Meier method, and a log-rank test was used to compare the possible prognostic factors between different groups. The Cox proportional hazard model was used to perform multivariable analysis of significant covariants.

**Results:**

A total of 211 LSCC patients were included, of which 164 (77.7%) were male and 47 (22.3%) were female. Mean age was 62.19 ± 8.328 years. A univariate analysis showed that seven factors including pathological differentiation, clinical stage, tobacco consumption, alcohol consumption, T stage, N stage, and concurrent chemoradiotherapy were correlated with survival (*P*<0.05). Cox proportional hazards regression analyses revealed that clinic stage (hazard ratio=3.100, p=0.048), pathological differentiation (hazard ratio = 2.538, p=0.015), alcohol consumption (hazard ratio = 8.456, p =0.004) were associated with OS in LSCC. Pathological differentiation (hazard ratio =5.677, p=0.000), alcohol consumption (hazard ratio =6.766, p=0.000) were associated with PFS in LSCC.

**Conclusions:**

Pathological differentiation, alcohol consumption, are independent prognostic factors and predictors of recurrence in LSCC. These factors could help inform guidelines for clinical treatment and prognosis.

## Introduction

Laryngeal squamous cell carcinoma (LSCC) is the second most common primary malignant tumor of the respiratory tract after lung cancer. It is, also the second most common primary epithelial malignant tumor of the head and neck. The age of onset of LSCC is mostly between 50 and 70 years. With a sex ratio of approximately 4:1, most LSCC patients are male (1). According to estimates by the American Cancer Society, in the United States, approximately 12,370 patients will be diagnosed with LSCC and 3750 of them will die from the disease in 2020 (2). Etiology has confirmed that smoking and drinking are related to the occurrence and development of LSCC, and the survival rate of smokers and drinkers is lower than that of non-smokers and non-drinkers (1, 3). Due to the increase in tobacco and alcohol consumption and occupational exposure to toxic substances like polycyclic aromatic hydrocarbons (PAH), the prevalence rate of LSCC has increased in recent years (4, 5).

The factors affecting the prognosis and survival of patients with LSCC can be classified into host, tumor, and treatment factors. The 5-year survival rate for patients with early LSCC is 70 to 90%; while for patients with advanced LSCC, it is only about 30%. Some published studies have stated that younger patients have better survival rates and prognosis than older patients (6, 7), but other studies observed that younger patients have higher risk of recurrence than older patients (8). Sex is another factor related to LSCC prognosis, with females appearing to have better prognosis than males (9). However, this trend may be due to other factors such as the uneven distribution of smoking habits between males and females. Malnutrition has also been identified as an independent prognostic factor of LSCC (10). Further, general condition of the patients, such as the existence of complications, can affect prognosis and survival. For example, pre-treatment hemoglobin levels were also found to be another factor affecting prognosis (11, 12). Regarding the immunological response, immunosuppressed patients seem to have a poor prognosis (13). The site of the primary tumor can also affect prognosis. According to the anatomical position, LSCC can be divided into supraglottic, glottic, and subglottic. In recent years, classification of LSCC as para-glottic LSCC has become controversial and has not been confirmed by the Union for International Cancer Control. Para-glottic LSCC originates in the laryngeal chamber and crosses the supraglottic region and glottic area. Supraglottic cancers have worse prognosis than glottic and subglottic cancers. This could be attributed to the fact that supraglottic cancers have a higher risk of lymph node metastasis (14). Clinical stage is another obvious prognostic factor (9). Increasing T and N stages could lead to higher risk of recurrence and poor prognosis (15). Distant metastases are also associated with poor survival (16). Patients with cervical lymph node metastasis had a worse prognosis than those without lymph node metastasis. Further, compared with highly differentiated LSCC, poorly differentiated LSCC usually has a higher risk of metastases (17). Finally, there are also several biomarkers, such as EGFR (18), WRAP53β, p16INK4a (19), estrogen receptor (ER-β)progesterone receptor (PR) (20), p53 (21, 22), and Bcl-2 (23) which have been linked with poor prognosis and lower survival rate.

The main treatments for LSCC are surgery, radiotherapy, and chemotherapy. Partial laryngectomy or total laryngectomy is feasible in early cases, and new laryngeal reconstruction is feasible in total or subtotal laryngectomy. Management of LSCC is particularly challenging due to the substantial functional morbidity and psychosocial impact of laryngectomy. Therefore, there is a need to find a balance between optimal tumor control and preserving organ function. While the efficacy of radiotherapy alone for early LSCC is similar to surgical treatment, the physiological function of the larynx can be preserved better by radiotherapy alone. When radiotherapy fails, salvage surgery is feasible. For middle and advanced LSCC, comprehensive treatments such as surgery, radiotherapy, and chemotherapy are the main treatments. Preoperative or postoperative radiotherapy can improve survival rate. The overall survival (OS) and progression free survival (PFS) of patients with negative margins have been shown to be better than those of patients with positive margins (24). The curative effect of surgical treatment has been reported to be better than that of radiotherapy alone (25).

Other factors such as HPV infection can also be pathogenic for LSCC (26). However, whether factors such as sex or age are involved in the prognosis of LSCC remain unclear and require further study (27). We performed a retrospective analysis to investigate the possible prognostic factors of LSCC, including sex, age, tumor location, clinical stage, pathological differentiation, tobacco consumption, and alcohol consumption. Our study could help inform clinical strategies for treatment and improve the survival rate and quality of life of patients.

## Methods

This study included patients with LSCC treated in our hospital from April 2011 to July 2019. The research was approved by the Ethics Committee of the First Bethune Hospital of Jilin University, and all participants provided informed consent. Inclusion criteria were as follows: 1) LSCC was confirmed by pathological diagnosis; 2) complete clinical history and informed consent was provided; 3) complete follow-up data were available; 4) In the early stage of LSCC, radical radiotherapy is performed, and postoperative radiotherapy or concurrent chemoradiotherapy is required. Exclusion criteria: 1) Patients with distant metastasis before treatment; 2) Patients whose histopathological type is not squamous cell carcinoma; 3) Patients who have not completed the treatment plan; 4) Patients without survival data. Patients were staged according to the American Joint Committee on Cancer (AJCC) cancer staging manual, 7th edition (28). We collected information on the following prognostic factors of selected patients: age, sex, smoking, drinking, stage, classification, and pathological differentiation.

Follow-up data which contained survival status, disease progression, recurrence, and death, were collected every 3 months. OS was defined as the time from the date of diagnosis to the date of death. PFS was defined as the time from diagnosis to disease progression or death (if no progression was reported before death) or the date of last follow-up. Recurrence is classified as local, regional, and distant metastasis.

Statistical analysis was performed using SPSS 26.0 (SPSS Inc., Chicago IL, USA). Quantitative data were presented as mean ± SD while qualitative data were presented by rate. The overall survival rate (OS) and progression-free survival rate (PFS) were estimated by Kaplan-Meier curve. Kaplan-Meier curves were compared according to age, sex, smoking, drinking, staging, classification, pathological differentiation and simultaneous radiotherapy and chemotherapy. The independent factors affecting mortality and progression (recurrence and metastasis) without metastasis were evaluated by Cox proportional hazard ratio model. The significant factors observed in the univariate Cox proportional hazard ratio model were gradually incorporated into the multivariate Cox proportional hazard ratio model, except that T period and N period were excluded because of multiple collinearity, the other factors gradually entered the multivariate Cox proportional risk ratio model. All statistical tests were two-sided. Differences were considered statistically significant at P values < 0.05.Results.

### Baseline Characteristics

For the duration of the study, we included patients admitted to our hospital from April 2011 to July 2019 and according to the exclusion criteria. Patient characteristics are shown in **Table 1**. Patients included 164 males (77.7%) and 47 females (22.3%), and mean age was 62.19 years (range 41–87). There were 167 patients (79.1%) with a history of tobacco consumption, while 141 of the patients (66.8%) had a history of alcohol consumption. Types of LSCC included supra-glottic (50.2%), glottic (43.6%), sub-glottic (2.4%), and para-glottic (3.8%). Most patients were stage T2 + T1 (71.1%), 28.9% were T3 + T4. More than a half of the patients (59.7%) were in N0 stage, 11.8% were in N1, 27.5% in N2, and 0.9% in N3. Nearly 30% of patients were at clinical stage I (26.1%), and more than 30% were at clinical stage IV (35.1%). Meanwhile, 20.9% and 79.1% of patients had low or high pathological differentiation, respectively. Most patients (63.5%) were treated with surgery and radiation 67 patients (31.8%) were accepted radiotherapy and surgery plus chemotherapy, while patients treated with radiotherapy only and radiotherapy plus chemotherapy were 2.4% and 2.4%, respectively.

**Table 1:** Summary of baseline characteristics.

		Frequency Percent/Mean ± SD
**Sex**		
	Male	164 (77.7%)
	Female	47 (22.3%)
**Age**		
	Mean ± SD	62.19 ± 8.328
**Tobacco**		
	Yes	167 (79.1%)
	No	44 (20.9%)
**Alcohol**		
	Yes	141 (66.8%)
	No	70 (33.2%)
**Type**		
	supra-glottic	106 (50.2%)
	glottis	92 (43.6%)
	sub-glottis	5 (2.4%)
	para-glottic	8 (3.8%)
**Clinic stage**		
	I	55 (26.1%)
	II	48 (22.7%)
	III	34 (16.1%)
	IV	74 (35.1%)
**T stage**		
	T1+ T2	150 (71.1%)
		
	T3+ T4	61 (28.9%)
		
**N stage**		
	N0+ N1	151 (71.6%)
		
	N2+ N3	60 (28.4%)
		
**pathological differentiation**		
	High	167 (79.1%)
	Low	44 (20.9%)
**concurrent chemoradiotherapy**		
	Yes	72 (34.1%)
	No	139 (65.9%)

### Overall Survival and Prognosis Factors of LSCC

**Figure 1:**
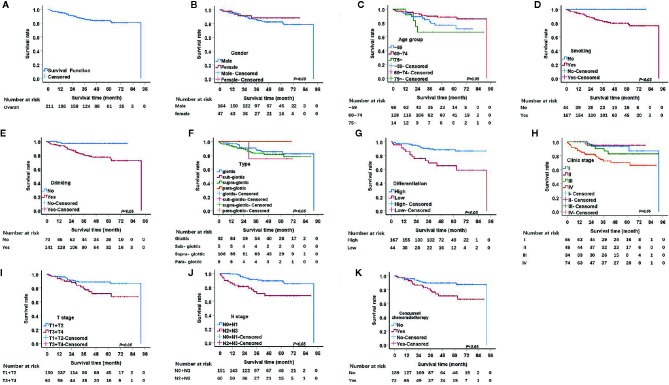
Kaplan.-Meier curves for overall survival (OS). **(A)** Kaplan-Meier curves for overall survival rate. **(B)** There was no significant differences between male and female, p>0.05. **(C)** There was no significant differences among age group, p>0.05. **(D)** The survival rate for tabacoo users was significantly lower than non-smokers, p=0.005. **(E)** The survival rate for those who used to drink was significantly lower than those did not, p=0.001. **(F)** There was no significant difference among group of type, p>0.05. **(G)** The survival rate for those with low pathological differentiation tumors was significantly lower than those were high, p=0.000. **(H)** The survival rate for patients with clinical stage IV was significantly lower than those with I, II, III, respectively, p=0.001. **(I)** The survival rate for those at stage T3 and T4 was significantly lower than those at T1 and T2, p=0.017. **(J)** The survival rate for those at stage N2 and N3 was significantly lower than those at N1 and N0, p<0.05. **(K)** The survival rate for those with concurrent chemoradiotherapy was significantly lower than those not, p<0.05.

The median follow-up period was 48 months. The 1, 3, and 5-year OS rates were 95.2%, 85.9%, and 83.5%, respectively. The univariate analysis demonstrated that seven factors, including pathological differentiation, clinical stage, tobacco consumption, alcohol consumption, T stage, N stage, and concurrent chemoradiotherapy were significantly associated with survival (P<0.05) (**Table 2**, **Figure 1**). The Kaplan Meier survival curves showed that patients with high pathological differentiation had a more favorable prognosis than those with lower pathological differentiation. The 1, 3, and 5-year OS rates of low and high pathological differentiation were88.4%, 69.2%, and 65.3% and 97%, 90.3%, and 88.4%, respectively. For patients with clinical stage I, the 1, 3, and 5-year OS rates were 100%, 93.5%, and 93.5% respectively, which were better than those with stage II, III and IV. We combined the groups with stage T1 and T2 in order to compare survival status with a group containing patients with T3 and T4 stages. For the T1 and T2 group, the 1, 3, and 5-year OS rates were 96.0%, 88.7%, and 88.7%, respectively, while for the T3 and T4 group they were 93.4%, 79.5%, and 71.9%, respectively.

**Table 2:** Survival rates and univariable analysis of Kaplan-Meier.

		Survival rate (%)			Log Rank(χ^2^)	P value
		1 year	3 year	5 year		
**Sex**					0.773	0.379
	Male	95.1	85.2	82.2		
	Female	95.6	88.0	88.0		
**Age**					5.371	0.068
	~59	94.2	82.1	76.8		
	60–74	96.1	89.6	88.3		
	75~	91.7	66.7	66.7		
**Tobacco**					7.773	0.005
	Yes	94.0	82.8	79.9		
	No	100	100	100		
**Alcohol**					11.197	0.001
	Yes	93.6	80.6	77.1		
	No	98.6	97.0	97.0		
**Type**					1.715	0.634
	supra-glottic	93.3	83.1	81.5		
	glottis	96.7	88.8	85.2		
	sub-glottis	100	75.0	75.0		
	para-glottic	100	100	100		
**Clinic stage**					16.507	0.001
	I	100	93.5	93.5		
	II	100	95.0	95.0		
	III	100	90.2	82.6		
	IV	86.4	72.5	70.2		
**T stage**					5.677	0.017
	T1/T2	96.0	88.7	88.7		
	T3/T4	93.4	79.5	71.9		
**N stage**					12.025	0.001
	N0/N1	99.3	91.6	89.5		
	N2/N3	84.9	71.1	68.0		
**Pathological differentiation**					13.943	0.000
	Low	88.4	69.2	65.3		
	High	97.0	90.3	88.4		
**Concurrent chemoradiotherapy**					7.505	0.006
	Yes	94.4	78.8	70.9		
	No	95.6	89.5	89.5		

We also merged stages N2, and N3 and compared them with stage N0, N1. The 1, 3, and 5-year survival rates of the N2 and N3 group were 84.9%, 71.1%, and 68.0%, respectively and that of N0 and N1 were 99.3%, 91.6%, and 89.5%, respectively. The 1, 3, and 5-year OS rates of concurrent chemoradiotherapy and radiotherapy only were 94.4%, 78.8%, and 70.9% and 95.6%, 89.5%, and 89.5%, respectively. The 1, 3, and 5-year OS rates of smokers were 94.0%, 82.8%, and 79.9%, respectively. Surprisingly, all patients who did not smoke survived. Regarding alcohol consumption, the 1, 3, and 5-year survival rates of patients with a history of alcohol consumption were 93.6%, 80.6%, and 77.1%, respectively, while those of patients without a history of alcohol consumption were 98.6%, 97.0%, and 97.0%, respectively.

T stage and N stage were excluded from the multivariable analyses due to multicollinearity. At the same time, radiotherapy and chemotherapy were excluded because they did not accord with the clinical practice. The remaining four variables were gradually introduced into the multivariate Cox proportional hazard model through the forward LR method. Results from the Cox regression analysis showed that clinic stage, pathological differentiation and alcohol consumption are independent prognostic factors of LSCC (**Table 3**). Patients with low pathological differentiation had a higher risk than those with high pathological differentiation (hazard ratios of 2.538 p=0.015). As for patients with clinic stage IV had a higher risk than those with clinic stage I (hazard ratios of 3.100, p=0.048). Further, compared with non-alcohol consumers, patients with a history of alcohol consumption were also at higher risk, with a hazard ratio of 8.456, p=0.004.

**Table 3:** Multivariable Cox proportional hazard models of mortality.

Covariate		HR (95%Cl)	P value
**Alcohol**		LR	
	Yes	8.456 (2.013,35.512)	0.004
	No	–	
**Clinic stage**			
	I	–	
	II	0.541 (0.099,2.965)	0.479
	III	1.759 (0.468,6.606)	0.403
	IV	3.100 (1.009,9.528)	0.048
**differentiation**			
	Low	2.538 (1.197,5.381)	0.015
	High	–	

### Factors Influencing PFS

**Figure 2:**
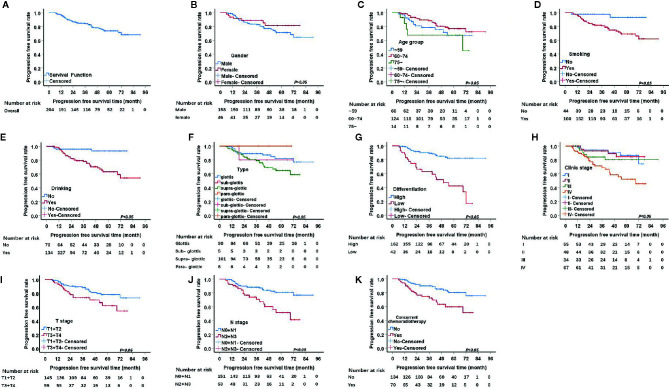
Kaplan-Meier curves of progression free survival (PFS) probability against time. **(A)** Kaplan-Meier curves for PFS. **(B)** There was no significant differences between male and female, p>0.05. **(C)** There was no significant differences among age group, p>0.05. **(D)** The PFS rate for tabacoo users was significantly lower than non-smokers, p=0.008. **(E)** The PFS rate for those who used to drink was significantly lower than those did not, p=0.000. **(F)** There was no significant difference among group of type, p>0.05. **(G)** The PFS rate for those with low pathological differentiation tumors was significantly lower than those were high, p=0.000. **(H)** The PFS rate for patients with clinical stage IV was significantly lower than those with I, II, III, respectively, p=0.000. **(I)** The PFS rate for those at stage T3 and T4 was significantly lower than those at T1 and T2, p=0.024. **(J)** The PFS rate for those at stage N2 and N3 was significantly lower than those at N1 and N0, p<0.05. **(K)** The PFS rate for those with concurrent chemoradiotherapy was significantly lower than those not, p<0.05.

From the 211 patients, seven patients were excluded because they died within a short period of time after admission, and therefore, PFS was not analyzed for them. Thus, we investigated the factors influencing PFS for the remaining 204 patients. The 1, 3, and 5-year PFS rate were 96.5%, 84.0%, 73.6%, respectively. The univariate analysis of PFS rendered similar results to those of OS. Seven factors, including pathological differentiation, clinical stage, tobacco consumption, alcohol consumption, T stage, and N stage, and concurrent chemoradiotherapy were significantly correlated with recurrence (P<0.05) (**Figure 2**). The Kaplan Meier survival analysis showed that patients with high pathological differentiation are more likely to have recurrence than patients with low pathological differentiation (P<0.05).

For patients with clinical stage I, the 1, 3, 5-year recurrence rates were 1.8%, 5.5%, 9.1%, respectively, which were better than those of patients with stage II, III and IV (P=0.000). For T1/2 group, the 1, 3, and 5-year PFS rates were 97.9%, 88.2%, and 78.1%, respectively, while for the T3/4 group the rates were 93.2%, 73.5%, and 62.0%, respectively.

The 1, 3, and 5-year PFS rates of the tobacco consumption group were 96.2%, 80.8%, and 69.1%, respectively, which were higher than the rates of the non-smoking group (P=0.008). Similar to the tobacco consumption group, the alcohol consumption group also had higher risk of recurrence than the non-drinking group (P=0.000). Thus, the 1, 3, and 5-year PFS rates were 96.9%, 78.1%, and 63.1% in the alcohol consumption group, respectively, and 95.7%, 95.7%, and 93.1% in non-drinking group, respectively.

It is generally believed that the N2/3 group is more likely to relapse than the N0/1 group, an idea which was confirmed by our data (P=0.000). The 1, 3, and 5-year PFS rates of the N0/1 group were 97.9%, 88.2%, and 80.9%, respectively, while those of the N2 and N3 group were 92.4%, 70.8%, and 51.6%, respectively.

The 1-, 3-, and 5-year survival rates of patients undergoing concurrent chemoradiotherapy and radiotherapy alone were 92.8%, 72.4%, 59.8%, and 98.5%, 89.9%, 80.4%, respectively (**Table 4**). We then performed a subtype analysis of concurrent chemoradiotherapy based on clinical stage in order to investigate whether the treatment decisions based on clinical staging affect recurrence. The results showed that, although there are no significant differences between all subtypes, the Kaplan Meier survival curve for patients with clinical stage IV showed that concurrent chemoradiotherapy led to a higher tendency of recurrence than radiotherapy only.

**Table 4:** Progression free survival rates and univariable analysis of Kaplan-Meier.

		Survival rate (%)			Log Rank(χ2)	P value
		1 year	3 year	5 year		
**Sex**					1.092	0.296
	Male	97.4	82.5	71.2		
	Female	93.5	88.8	81.2		
**Age**					4.389	0.111
	~59	95.4	78.5	67.0		
	60–74	98.4	88.3	76.9		
	75~	84.4	67.5	67.5		
**Tobacco**					6.968	0.008
	Yes	96.2	80.8	69.1		
	No	97.7	97.7	93.1		
**Alcohol**					14.229	0.000
	Yes	96.9	78.1	63.1		
	No	95.7	95.7	93.1		
**Type**					6.616	0.085
	supra-glottic	95.0	79.0	64.7		
	glottis	97.7	89.0	82.1		
	sub-glottis	100	80.0	80.0		
	para-glottic	100	100	100		
**Clinic stage**					18.619	0.000
	I	98.1	94.2	86.6		
	II	100	92.8	84.5		
	III	100	84.1	80.3		
	IV	91.0	68.9	52.1		
**T stage**					5.131	0.024
	T1+T2	97.9	88.2	78.1		
	T3+T4	93.2	73.5	62.0		
**N stage**					12.157	0.000
	N0+ N1	97.9	88.2	80.9		
	N2+N3	92.4	70.8	51.6		
**Pathological differentiation**					32.941	0.000
	Low	87.9	62.9	42.3		
	High	98.7	89.4	82.2		
**Concurrent chemoradiotherapy**					10.895	0.001
	Yes	92.8	72.4	59.8		
	No	98.5	89.9	80.4		

*Due to the limitation of our retrospective analysis, the 5 years survival rate of female, 75~, No tobacco history, Low pathological differentiation, stage Ⅱ and Ⅲ cannot be reached.

**The survival rate of group sub-glottis, para-glottic cannot be reached for limited sample size.

Next, we performed a Cox regression analysis to analyze the factors which reached statistical significance in the univariate analysis. Because of multicollinearity, T and N stages were excluded, and concurrent chemoradiotherapy was also excluded because it is not in line with real clinical practices. Smoking, drinking, clinical stages, and differentiation were gradually introduced into the multivariate Cox proportional hazard model through the forward LR method. The Cox regression analysis showed that pathological differentiation, alcohol consumption are independent disease progression factors of LSCC (**Table 5**). Those with low pathological differentiation had higher risk than those with high pathological differentiation (hazard ratios of 5.677, p=0.000). Compared with non-alcohol users, alcohol consumers had a higher risk, with a hazard ratio of 6.766, p=0.000.

**Table 5:** Multivariable Cox proportional hazard models of recurrence.

Covariate		HR (95%Cl)	P value
**Alcohol**			
	Yes	6.766(2.403, 19.051)	0.000
	No	–	
**differentiation**			
	Low	5.677(3.085, 10.444)	0.000
	High	–	

## Discussion

The prognosis of LSCC can be predicted by multiple factors, which can be divided into host, tumor, and treatment factors. In this study we assessed the influence of the above risk factors for the prognosis and recurrence of patients with LSCC. We report a 5-year OS and PFS of 83.5% and 73.6%, respectively, which are better than those reported in the literature (24). This could be due to improved diagnostic methods and better treatment. Otherwise, given that most of our patients had early stage LSCC, this could be attributed to an improvement in patients’ health awareness and the application of multiple examination methods (fiber laryngoscope, electronic laryngoscope, etc.)

Many factors have been reported to affect the prognosis of LSCC patients, such as age, race, smoking and so on. Sex has also been reported to be a prognostic factor for LSCC patients, and the prognosis of female patients is significantly better than that of male patients (9). But the conclusions from different studies are controversial. The univariate analysis results of this study showed that there was no statistical difference in the effect of gender on OS and PFS of LSCC patients. This is consistent with the findings of Walasek et al. (29). This may be related to the decreasing smoking rate among male patients and the increasing number of female smokers. In addition, age is also a prognostic factor affecting the survival. In the past, most scholars believed that younger patients had better survival than older patients (6, 8). This may be related to the better physical condition of the younger patients. However, it has also been found that younger patients have less differentiated tumors, usually poorly or undifferentiated, with higher rates of recurrence and metastasis, which may lead to lower survival rates. In this study, patients were divided into three groups according to their age groups, and the differences in OS and PFS of patients in different age groups were observed. The results did not show the differences in survival of LSCC patients in different age groups. In this study, most of the patients were middle-aged and elderly patients, with an average age of 62.19 years old. The age gap between the patients was relatively small, which may be the reason for this result.

Notably, unhealthy living habits also affect the occurrence, progression, and prognosis of LSCC. There is evidence that greater cigarette and alcohol consumption have an impact on the incidence and prognosis of LSCC (30, 31). However, other studies have shown different results. For example, Zhang et al. (24) showed that smoking and drinking have no effect on OS and PFS of LSCC. In our study, 167 (79.1%) patients had a history of tobacco consumption and 141 patients (66.8%) had a history of alcohol consumption. The univariate analysis showed that tobacco and alcohol are linked with recurrence and survival, and alcohol is an independent risk factor for OS and PFS. These results are consistent with the existing literature (3, 32). This suggests that smoking and drinking are important reasons for the poor prognosis of LSCC patients after radiotherapy, and lifestyle changes may become an important way to prevent the occurrence of LSCC and improve the prognosis. According to the SEER data, the 5-year survival rate of patients with LSCC varies according to the location of the primary tumor. For example, the 5-year survival rate of glottic cancer is higher than that of supraglottic cancer (6). Patients with supraglottic carcinoma have a higher recurrence rate, which may be related to their susceptibility to lymph node metastasis (8). Although the data showed that supraglottic carcinoma did not cause poor prognosis, the effect of anatomical location on LSCC should not be ignored. Consistently, no differences in survival rates were found among patients with tumors at different anatomic sites in the center. However, we observed that patients with glottic cancer had a more favorable clinical stage than patients with supraglottic cancer. As recommended by the guidelines, we have adopted a more aggressive treatment strategy for patients with supraglottic cancer. Better tumor control associated with intensive treatment may account for a similar prognosis in patients with glottic cancer. According to a multi-center study within the International Head and Neck Cancer Epidemiology (INHANCE) consortium, tumor stage is a positive predictor of cancer recurrence in HNC patients (33). Patients with advanced LSCC have an unfavorable prognosis (34). Most of our patients were stage IV (35.1%), and their 5-yearOS and PFS were 70.2% and 52.1%, respectively. Although the univariate analysis showed that both OS and PFS of patients at stage IV were much lower than those of patients at other stages, after excluding T and N stages, the multivariate Cox regression analysis showed that clinical stage was an independent risk factor for survival while it was not an independent risk factor for recurrence of LSCC. Extensive evidence shows that tumor size and lymph node metastasis are important factors affecting the survival and recurrence rates of patients with LSCC (35). In a Danish study of 5001 people, Nina et al. (15) found that increased T stage was a risk factor for recurrence of glottic cancer. Johansen et al. (9) obtained a similar result, and showed that T stage and N stage have a significant effect on the prognosis of LSCC. In our study, T stage was an prognostic factor in the univariate analyses. We combined T1 and T2 groups and compared them with T3 and T4 groups. Univariate analysis showed that group T1/2 have a better prognosis than group T3/4, with 5 years OS and PFS of 88.7% and 78.1%, respectively. Regarding N stage, we merged the N0 stage and N1 stage into one group in order to compare the survival and recurrence with that of group N2 and N3. We observed that N stage has the same effect as T stage on OS and PFS. T and N stages seem to have become a recognized factor affecting prognosis (36). The results of this study were basically similar to the previous mainstream theories, which reflected the consistency of the influence for tumor stage on prognosis in different countries and regions. Though it is generally accepted that distant metastasis could cause unfavorable prognosis (16), the effect of M stage on prognosis could not be elucidated in this study because all patients were in stage M0.

The degree of tumor differentiation is linked with the survival and recurrence rate of patients with LSCC, with poorly differentiated cancers usually having a higher rate of metastatic disease compared with well-differentiated cancers (17). In this study, we merged the moderate- and well- differentiated cases of LSCC into one group and compared it with the group of poorly differentiated LSCC. Results showed that those with poorly differentiated LSCC had an unfavorable prognosis and higher recurrence rate in the univariate analysis. Moreover, the multivariate analysis demonstrated that differentiation is also an independent risk factor for survival and recurrence rate. Our results are consistent with the conclusion of Zhu et al. (37). However, the limitation of this study is that we were unable to assess the impact of moderate differentiation on the prognosis and recurrence of LSCC. In view of the effect of tumor differentiation on patient prognosis, the use of tumor stage alone as a criterion for treatment selection seems to be limited. The toxicity of more intensive treatment to highly differentiated tumors should be concerned. Our study provides a reference for the treatment of patients with highly differentiated tumors, suggesting that the degree of tumor differentiation should also be a reference factor for treatment selection. In order to avoid unnecessary injury caused by overtreatment, it may be possible to treat highly differentiated tumors by downgraded treatment. In addition, the choice of treatment may also be one of the reasons that affect the prognosis of patients. In a randomized controlled trial of 547 patients, Forastiere et al. (25) found no difference in survival between the radiotherapy alone group and the concurrent chemoradiotherapy group. Most of our patients (95.3%) accepted surgery either with concurrent chemoradiotherapy or without it, which might be the reason behind the high survival rate reported in our study. The univariate analysis showed that patients with concurrent chemoradiotherapy have more unfavorable prognosis and shorter PFS, which seems to contradict logic and is also in disagreement with the existing literature. However, according to the guidelines, concurrent chemoradiotherapy is recommended only for patients with advanced tumors. Therefore, the difference in baseline of patients in different treatment groups may be the reason for the different prognosis. Of course, the high adverse reactions of concurrent chemoradiotherapy should not be ignored, and it is urgent to develop drugs with less side effects.

Due to the retrospective design and small sample size of this study, our data did not include surgical margins, occupational exposure, or HPV infection. Therefore, we could not measure the impact of these factors on the incidence and prognosis of LSCC. However, we did analyze other factors that may affect the survival and recurrence rate of patients with LSCC, including patient, clinical and treatment factors. In this study we identified alcohol consumption and pathological differentiation as independent predictors of os for LSCC. Alcohol consumption, pathological differentiation and clinic stage were identified as independent predictors for os. Patients with a history of alcohol consumption and poor differentiation had a lower survival rate and were more prone to recurrence. There was no significant difference in OS and PFS between patients with concurrent radiotherapy and patients with radiotherapy alone, suggesting the importance of downgrading therapy in LSCC patients. In order to improve the survival rates of patients with LSCC, the importance of pathological differentiation, alcohol consumption and clinic stage on prognosis must be emphasized in the context of diagnosis and treatment.

## Data Availability Statement

The original contributions presented in the study are included in the article/supplementary materials. Further inquiries can be directed to the corresponding authors.

## Author Contributions

Conceptualization, XJ and YX; software, investigation, HHW, QHZ, ZZZ, QZ; resources, YYZ; writing-original draft preparation, HHW, QHZ, QZ, SYL, ZJL; writing-review and editing, LBM, YX, and XJ; funding acquisition, XJ. All authors contributed to the article and approved the submitted version.

## Funding

This work was supported by the National Natural Science Foundation of China (grant number 81570344), National Key R&D Program of China (grant number 2017YFC0112100), the Education Department Foundation of Jilin Province (grant number JJKH20201036KJ), the Health and Family Planning Commission of Jilin Province Foundations (grant number 2016Q034 and 2017J11), the Fundamental Research Funds for the Central Universities of Jilin University, and the Jilin Provincial Science and Technology Foundations (grant number 20180414039GH and 20190201200JC).

## Conflict of Interest

The authors declare that the research was conducted in the absence of any commercial or financial relationships that could be construed as a potential conflict of interest.

## Abbreviations

EGFR, Epidermal growth factor receptor; HPV, Human papilloma virus; LSCC, Laryngeal squamous cell carcinoma; OS, Overall survival (OS); PFS, Progression free survival.
